# The kinetics of inhibitor production resulting from hydrothermal deconstruction of wheat straw studied using a pressurised microwave reactor

**DOI:** 10.1186/1754-6834-7-45

**Published:** 2014-03-29

**Authors:** Roger Ibbett, Sanyasi Gaddipati, Darren Greetham, Sandra Hill, Greg Tucker

**Affiliations:** 1 BBSRC Sustainable Bioenergy Research Centre, University of Nottingham, Sutton Bonington Campus, Loughborough, Leicestershire LE12 5RD, UK

**Keywords:** Biomass, Hydrothermal, Deconstruction, Kinetics, Inhibitors, Microwave, Wheat straw

## Abstract

**Background:**

The use of a microwave synthesis reactor has allowed kinetic data for the hydrothermal reactions of straw biomass to be established from short times, avoiding corrections required for slow heating in conventional reactors, or two-step heating. Access to realistic kinetic data is important for predictions of optimal reaction conditions for the pretreatment of biomass for bioethanol processes, which is required to minimise production of inhibitory compounds and to maximise sugar and ethanol yields.

**Results:**

The gravimetric loss through solubilisation of straw provided a global measure of the extent of hydrothermal deconstruction. The kinetic profiles of furan and lignin-derived inhibitors were determined in the hydrothermal hydrolysates by UV analysis, with concentrations of formic and acetic acid determined by HPLC. Kinetic analyses were either carried out by direct fitting to simple first order equations or by numerical integration of sequential reactions.

**Conclusions:**

A classical Arrhenius activation energy of 148 kJmol^−1^ has been determined for primary solubilisation, which is higher than the activation energy associated with historical measures of reaction severity. The gravimetric loss is primarily due to depolymerisation of the hemicellulose component of straw, but a minor proportion of lignin is solubilised at the same rate and hence may be associated with the more hydrophilic lignin-hemicellulose interface. Acetic acid is liberated primarily from hydrolysis of pendant acetate groups on hemicellulose, although this occurs at a rate that is too slow to provide catalytic enhancement to the primary solubilisation reactions. However, the increase in protons may enhance secondary reactions leading to the production of furans and formic acid. The work has suggested that formic acid may be formed under these hydrothermal conditions via direct reaction of sugar end groups rather than furan breakdown. However, furan degradation is found to be significant, which may limit ultimate quantities generated in hydrolysate liquors.

## Introduction

Physicochemical pretreatments are required to increase the efficiency of enzyme hydrolysis of the polysaccharide fraction of lignocellulosic biomass, in order to liberate fermentable sugars for production of ethanol. The lignocellulosic cell wall consists of an assembly of cellulose fibrils, sheaved in a layer of hemicellulose, which acts as an interface with a surrounding network of lignin [1]. This highly recalcitrant structure must be subjected to controlled deconstruction in order to increase the accessibility and reactivity of the cellulose fibrils, which is required to maximise the rate and yield of saccharification to liberate glucose. Hydrothermal processing is one of the most promising deconstruction methods for lignocellulosic biomass, carried out in water at high temperatures and pressures, where the cell wall is disrupted by hydrolysis of the hemicellulose component coupled with degradation and temporary liquefaction of the lignin component [2,3]. The process is particularly attractive as it has low chemical demand and leads to a deconstructed product with high cellulose digestibility. The technology has been demonstrated successfully at pilot-scale and has the potential for large-scale operation, although some disadvantages still need to be overcome before full commercialisation is realised. Most significantly, the reaction conditions lead to the formation of aromatic, furanic and organic acid by-products, which inhibit yeast fermentation [4]. Also, the need for operation at high temperatures leads to a high energy demand, with the corresponding engineering difficulties in containing water at high temperature and pressures. A better understanding of the mechanisms and chemistry of hydrothermal deconstruction of lignocellulosic biomass is therefore required, in order to establish process conditions which minimise inhibitor production, whilst maximising the desirable deconstruction reactions and minimising energy consumption.

Central to the improvement in understanding of hydrothermal processes is the need for fundamental information on the kinetics of the various pathways associated with the deconstruction of lignocellulosic biomass. This includes the reactions involving the disassembly of separate hemicellulose and lignin fractions in the cell wall and the degradation reactions within these fractions leading to formation of inhibitory by-products. Information at this basic level will be helpful both in the design of processing technologies and also in the selection of operational times, temperatures and concentrations. However, access to controlled kinetic data is challenging, as typical small-scale reactors used for research investigations require significant heat-up times, so measurements may suffer from non-isothermal conditions [5]. This difficulty can be partly circumvented by controlled overheating and then stabilisation [6], injection of preheated chemicals [7], or by correction for the heat-up time [8], or by working with a severity factor scale based on non-isothermal measurements [9]. Studies of the progress of hydrothermal deconstruction reactions of biomass under different conditions have been reported, which allude to more fundamental kinetic parameters [10-13]. However, the underlying rate constants and activation energies for reactions concerned with inhibitor generation are reported more rarely [5,14,15], without which it is difficult to make effective predictions or carry out modelling.

In this new study we have made use of a laboratory microwave synthesis reactor with the capability to apply controlled heating to samples of biomass in pressurisable vessels. Other studies of microwave processing of biomass have been reported, either for pyrolysis [16], extraction [17] or chemical conversion [18]. Microwave-assisted kinetic studies of biomass pyrolysis have also been reported [19] and also aqueous alkali-assisted pretreatment at ambient pressure [20]. A kinetic study of microwave-assisted synthesis of furfural from xylose has been reported using liquid water and water/organic mixtures at elevated pressure [21].

The use of microwave dielectric heating at 2.45 GHz, rather than conductive or steam heating, is not considered to influence the fundamental reactions taking place in the biomass material [17], but offers a method for uniform volumetric delivery of thermal energy by virtue of the dielectric properties of water, which is present in excess within the reaction mixture. This is distinct from studies where specific microwave enhancements have been postulated [22]. The reactor can be programmed to heat the sample rapidly, with accurate temperature feedback, then to maintain a precise set temperature for a selected time and then to cool the sample rapidly by forced air flow to halt the progress of reactions. The reaction products for each experiment time can then be collected and analysed to build up kinetic profiles for concentrations of relevant species. Direct gravimetric measurements of total solubilised species have been made in order to link with classical studies of biomass conversion reactions, for example associated with wood pulping [23]. The collection of gravimetric data has also allowed a simplification of interpretation and kinetic modelling, and has also allowed comparisons with reaction severity equations commonly used for biomass pretreatment for ethanol production [11]. We have demonstrated how the hydrothermal deconstruction of wheat straw can be interpreted using standard or modified kinetic models, to gain understanding of the interrelationships between kinetic parameters and hence chemical pathways between different solubilised species. These associations will also assist in understanding the morphological relationships between cell wall components and changes occurring during deconstruction. A better appreciation of the origins of formation of inhibitor compounds will also assist in programmes concerned with selection of optimal feedstock phenotypes, where cellulose structure and composition may be manipulated by breeding or genetic modification.

## Results and discussion

### Kinetics of primary hydrothermal reactions

#### Global solubilisation

**Figure 1:**
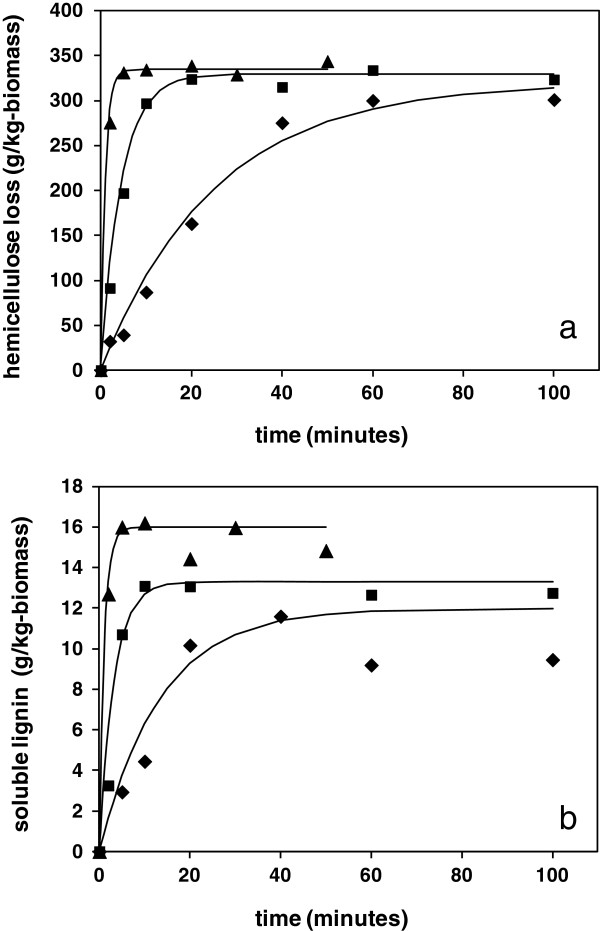
**Kinetics of solubilisation of wheat straw under hydrothermal conditions. a)** Total solubilised mass; and **b)** soluble lignin in hydrolysate. ♦, 180°C; ■, 200°C; ▲, 220°C.

The rates of solubilisation of the wheat straw at the selected reaction temperatures of 180, 200 and 220°C are shown from the gravimetric profiles in Figure 1a. The combined mass loss from the depolymerisation and solubilisation of all species therefore provides an unambiguous measure of the extent of deconstruction of wheat straw, so the derived kinetic parameters can be used to describe a global reaction ordinate, as discussed later. Around 5% of the sample mass was found to be easily extractable in water, consisting of loosely bound sugars, soluble inorganic material and other residues. This constituted a background which was subtracted prior to kinetic analysis. After subtraction, at all the studied process temperatures the kinetic profiles reached an asymptote of around 35 wt% of total biomass. The non-polar waxy fraction of wheat straw accounts for approximately 4% of total mass, from Table 1, which may form a melt dispersion during the hydrothermal treatment but is expected to re-precipitate on cooling and should therefore not contribute to the mass loss [24].

**Table 1:** Composition of straw biomass

**Component**	**g/kg-biomass**
Cellulose	395
Hemicellulose	293
Lignin	230
Wax	40
Ash	45
	**g-sugar/kg-biomass**
Glucose	436
Xylose	192
Arabinose	28
Mannose	7
Galactose	8

The kinetic profiles for the total mass loss could be successfully fitted to a first order kinetic equation (1), overlayed on the data in Figure 1a, where S_t_ is the mass removed at time t, in units of g/kg-original dry mass, S_inf_ is the mass removed at infinite time and k is the rate constant. The fitted rate constants for each temperature are summarised in Table 2, from which the activation energy (E) and pre-exponential factor (A) could be derived, via Arrhenius analysis using equation (2).

(1) $$S_{t}=S_{\inf}\left(1-e^{-\mathrm{kt}}\right)$$

(2) k=Ae−ERT

(3) $$R_{0}=te^{\left(T_{r}-T_{b}\right)/14.75}$$

**Table 2:** Fitted rate constants and activation energies for primary hydrothermal solubilisation reactions of wheat straw

**Temperature (°C)**	**Total solubilisation**		**Hemicellulose solubilisation**		**Lignin solubilisation**	
	**k**_**s **_**(min**^**−1**^**)**	**R**^**2**^	**k**_**h **_**(min**^**−1**^**)**	**R**^**2**^	**k**_**l **_**(min**^**−1**^**)**	**R**^**2**^
180	0.040 (0.012)	0.987	0.040 (0.01)	0.988	0.074 (0.029)	0.974
200	0.221 (0.033)	0.994	0.219 (0.034)	0.990	0.31 (0.055)	0.950
220	0.982 (0.099)	0.999	0.993 (0.105)	0.999	1.12 (0.295)	0.990
Arrhenius analysis						
Es (kJmol^−1^)	148 (21, 1.65)	0.999	149 (16, 1.23)	0.998	126 (12, 0.90)	0.999

First or single number in brackets, ±95% confidence limits of parameter estimation; second number in brackets, ± standard error of parameter estimation.

The knowledge of the Arrhenius parameters allows a prediction of the extent of progress of the collective deconstruction reactions of wheat straw under any combination of time and temperature. From this it is possible to revisit the concept of reaction severity (the reaction ordinate), based on the approach followed by Overend and Chornet from earlier work [23,25]. This assumes that, in bulk, the thermally activated depolymerisation-solubilisation reactions in woody materials follow pseudo-first order kinetics, so the global extent of reaction is related to the severity parameter (R_o_), which is defined as an empirical function of time and temperature in equation (3), where T_r_ is the reaction temperature and T_b_ is a reference temperature (usually taken as 100°C). For comparison, the classical function ln(k.t) can be calculated directly from equation (2) using the Arrhenius parameters of the current study, which correlates linearly with the function ln(R_o_) derived from equation (3). However, the correlation is offset as a result of a different choice of Arrhenius activation energy for the original equations referenced by Overend and Chornet and Chum and others, of 113 kJmol^−1^, compared to the value of 148 kJ/mol found from the current study [23,25]. Future studies might make use of this higher activation energy, for calculation of the classical function (k.t) or ln(k.t), if this is more predictive of solubilisation behaviour of arable plant residues under hydrothermal conditions. Further kinetic studies would be required to confirm if this higher activation energy for deconstruction is appropriate for other biomass residues or energy crops. An activation energy for solubilisation of 130 kJmol^**−**1^ was found for the autohydrolysis of corn cobs using a slow heat-up reactor, with delayed onset of sampling [5]. The current microwave protocol achieved more genuine isothermal conditions, accurately recorded by *in situ* temperature measurement.

### Lignin solubilisation

Approximately 6 to 8% of the total lignin content of the straw was solubilised in the reaction liquor under the hydrothermal conditions of this study. Depolymerisation is believed to be due to scission of the predominant beta-aryl ether bonds between phenylpropanoid units, which liberates fragments sufficiently small and with sufficient polarity to achieve water solubility [26]. All solubilised aromatic fragments derived from lignin are expected to have similar chromophores, including vanillic, cumaric, cynamic species, and so on. The UV data therefore provides a total measure of lignin-derived inhibitor concentration, which avoids the need for chromatographic determination of each specific molecular species. The concentration profiles at each reaction temperature could be fitted to a first order kinetic equation, analogous to equation (1), with a defined final concentration, as shown in Figure 1b, with rate constants and derived activation energy also summarised in Table 2. The late data points showed some evidence of lignin recondensation, more prevalent at lower liquor ratio as described later, which was accounted for by a constant factor. Given this additional influence, the rate constants determined for lignin solubilisation were slightly higher than that of global solubilisation and the corresponding activation energy of 135 kJmol^−1^ was slightly lower, although consistent with hydrolysis type reactions. However, with lignin it is also possible that in hydrophobic regions the aryl-ether bonds will undergo thermally induced homolytic rupture [27]. Scissions in these less accessible lignin regions would be more likely to be followed by recondensation of the reactive species, resulting in insolubility as described in earlier studies [3,10]. The lignin fragments extracted into the liquor may therefore be from the more hydrophilic water accessible regions of the lignin network, which might be more closely associated with the hemicellulose components of the cell wall. From the kinetic profiles, the final amount of solubilised lignin increased slightly with increasing reaction temperature from 180 to 220°C, which may be a result of an improvement in accessibility through higher water activity.

### Hemicellulose solubilisation

The majority of the cellulose component of the cell wall is retained in the solid residue under these hydrothermal conditions, although a minor amount of glucan may be depolymerised and will therefore make a small contribution to the mass loss [23]. The major proportion of solubilised mass is a result of the hydrolysis of the arabinoxylan polysaccharides in the cell wall, which are extracted into the aqueous liquor after sufficient bond scissions of the polymer backbone have occurred to reduce the molecular weight below the solubility threshold. This leads to the creation of a range of soluble xylose monomers and oligomers, together with minor amounts of other hemicellulose sugars, with various functionalities. Discounting the minor contribution from cellulose, the difference between the values for total gravimetric mass loss and that of solubilised lignin provided a measure of the rate of solubilisation of all hemicellulose species. The resultant profiles were fitted successfully to the first order kinetic equation (1), with the corresponding activation energy for wheat straw under these hydrothermal conditions found to be 149 kJmol^−1^. This was only slightly different from that of total biomass solubilisation and is consistent with published values for hydrolysis of other hemicellulose materials [3].

The gravimetric protocol for hemicellulose solubilisation provides a less complex alternative to the full compositional analysis of the solid residue at each time point. Also, the gravimetric measurement avoids the complication of identification of both oligomers and monomers in the hydrothermal liquors in subsequent kinetic analyses, or the uncertainty in defining the oligomer molecular weight at the point of liberation from the biomass solid. The relationship between the rate constant for mass removal and that of individual polysaccharide bond scission has been considered mathematically for a model cellulose, where a constant factor was applied, approximately proportional to the degree of polymerisation of the soluble fragments [28]. However, a realistic heterogeneous model would be more complex, as liberation of hemicellulose fragments from the insoluble cell wall will require other energy input, for example to break hydrogen bonds and to allow conformational movement of the fragments away from the cell wall surfaces. The activation energies for oligomer production by weight loss and individual monomer production by bond scission may therefore be different, as explored in the next section.

### Kinetics of secondary hydrothermal reactions

#### Generation of furans

**Figure 2:**
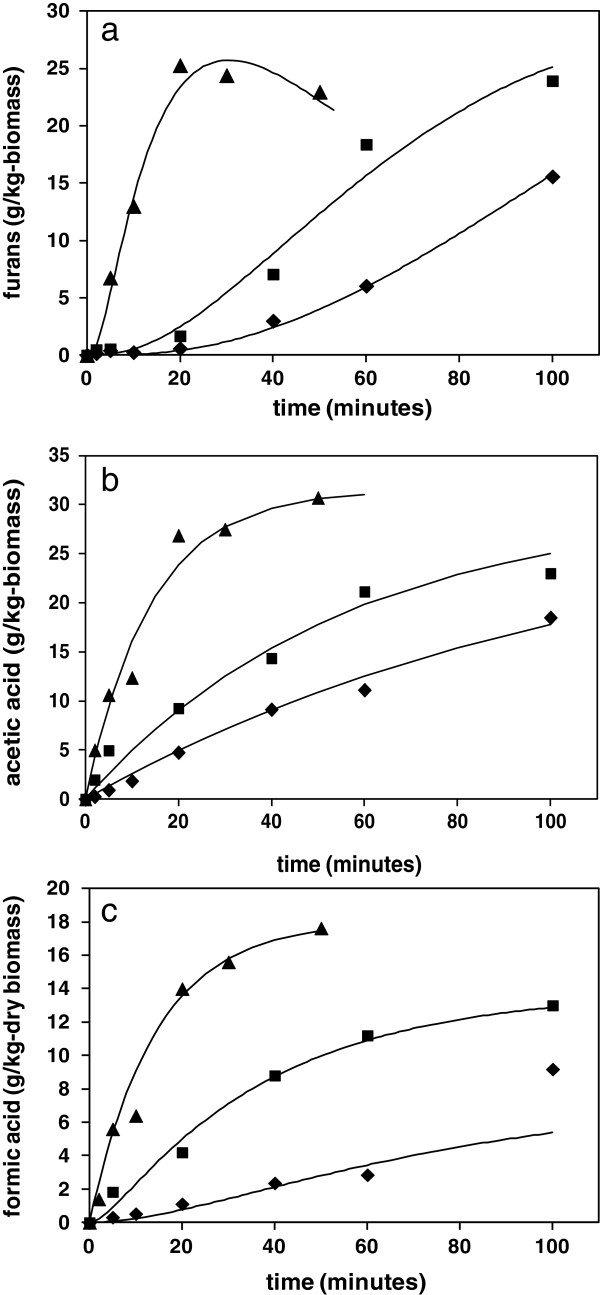
**Kinetics of generation of inhibitor compounds in hydrolysate liquors following hydrothermal processing of straw. a)** Furans; **b)** acetic acid; and **c)** formic acid. ♦, 180°C; ■, 200°C; ▲, 220°C.

The hemicellulose oligomers liberated into solution undergo continuing hydrolysis to form a variety of pentose monomers, which then undergo dehydration reactions to give furfural as a major product, which then further degrades to a variety of molecules, including various organic acids and condensation products [29]. The UV analysis method provided a fast, reliable measure of the evolution of total furans with time, as shown for wheat straw at the three reaction temperatures in Figure 2a. A minor amount of cellulose may be solubilised under hydrothermal conditions, although other soluble C6 sugars may be derived from minor glucan or galactan constituents of hemicellulose. These C6 sugars will undergo dehydration to form 5-(hydroxymethyl)furfural (HMF), which will be detected by UV within the total furan response. For subsequent kinetic analyses all furanic species are assumed to be derived from hemicellulose. Separate analyses by HPLC confirmed that concentrations of HMF were present at a constant 7% proportion of furfural in the hydrolysates from this study, and it was therefore assumed that both C6 and C5 dehydration followed the same rate behaviour.

**Figure 3:**
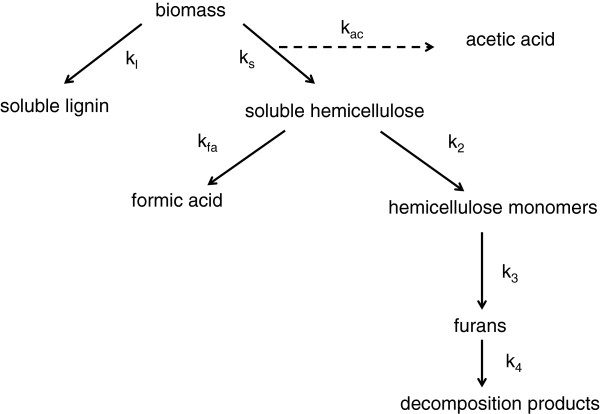
Kinetic scheme for hydrothermal reactions of straw biomass.

For this study a scheme of linked first order reactions was proposed to account for the production and degradation of furanic species, outlined schematically in Figure 3, where the individual first order rate constants are shown for each step in the pathway. Various assumptions are implicit in the scheme, which have been introduced to improve the manageability of analysis, hopefully without sacrificing chemical reality. Firstly, it was assumed that there is a very low statistical chance of sugar monomers being directly released by endwise loss from polymer chains held in the cell wall. The apparent mono-exponential kinetics of initial hemicellulose solubilisation from the cell wall (rate constant k_s_) also suggested that it was only necessary to consider one initial reacting species and hence a single initial reaction step. Also, it was considered that only sugars converted to monomer form (rate constant k_2_) could undergo subsequent conversion to furanic species (k_3_), via dehydration mechanisms [29]. Initially it was assumed that all hemicellulose followed the furan pathway, although in the later discussion it is postulated that some might react to give formic acid. Degradation of furans via all possible pathways was approximated by a single rate constant (k_4_).

The reaction scheme is represented mathematically by a set of first order differential rate equations, shown as equations (4, 5, 6 and 7), where C, H, M and F are the concentrations of unreacted hemicellulose polymer, soluble hemicellulose oligomers, sugar monomers and furans, respectively.

(4) $$\frac{\mathrm{dC}}{\mathrm{dt}}=-k_{s}C\;$$

(5) $$\frac{\mathrm{dH}}{\mathrm{dt}}=k_{s}C-k_{2}H-\left[k_{\mathrm{fa}}H\right]$$

(6) $$\frac{\mathrm{dM}}{\mathrm{dt}}=k_{2}H-k_{3}M\;$$

(7) $$\frac{\mathrm{dF}}{\mathrm{dt}}=k_{3}M-k_{4}F$$

The evolution of the concentrations of the different species with time were calculated by numerical integration of these coupled ordinary differential equations (ODEs). Errors between calculated and experimentally measured furan concentrations were minimised by adjustment of the k_2_, k_3_ and k_4_ rate constants, with the k_s_ rate constant fixed initially for each minimisation, as determined from the kinetics of gravimetric solubilisation of hemicellulose. The initial amount of hemicellulose polysaccharide (C_o_) was taken from the straw compositional assay of 235 g/kg-dry biomass, as sugar monomer, with the molecular weights of furfural and xylose used for mole conversions. The mass balance of all species was confirmed over time. The resulting fitted concentration profiles are superimposed on the experimental furan data in Figure 2a, with corresponding rate constants from the minimisation summarised in Table 3. The differential rate equation (5) also includes an optional term for generation of formic acid directly from soluble oligomers, which is considered separately below.

**Table 3:** Fitted rate parameters and activation energies for secondary hydrothermal reactions of wheat straw

**Liquor ratio**	**Parameters**	**Furans**					**Fur-sim**	**Acetic acid**		**Ac-sim**	**Formic acid**
		**C**_**o **_**(g/kg)**	**k**_**s **_**(min**^**−1**^**)**	**k**_**2 **_**(min**^**−1**^**)**	**k**_**3 **_**(min**^**−1**^**)**	**k**_**4 **_**(min**^**−1**^**)**	**R**^**2**^	**C**_**inf **_**(g/kg)**	**k**_**ac **_**(min**^**−1**^**)**	**R**^**2**^	**K**_**f **_**(min**^**−1**^**)**
10:1		Kinetic modelling (not including formic acid)									
	180°C	235	0.040	0.019	0.0016	0.0021	0.994	31 (12)	0.0076 (0.0051)	0.993	0
	200°C	235	0.219	0.060	0.0053	0.0138	0.978	30 (7.2)	0.0214 (0.011)	0.983	0
	220°C	235	0.993	0.211	0.0183	0.0684	0.984	32 (6.6)	0.072 (0.033)	0.972	0
10:1		Arrhenius analysis (not including formic acid)									
	E (kJmol^**−**1^)		149 (16, 1.2)	112 (68, 4.9)	113 (49, 3.8)	166 (78, 6)			104 (93, 7.4)		
	R^2^ of Arrh fit		0.999	0.997	0.998	0.998			0.995		
4:1		Kinetic modelling (not including formic acid)									
	200°C	235	0.219	0.12	0.017	0.031	0.997	28 (2.8)	0.040 (0.009)	0.992	0
10:1		Kinetic modelling (including formic acid)									
	180°C	235^a^	0.04^a^	0.010	0.0037	0.0021^a^	0.994				0.0018
	200°C	235^a^	0.219^a^	0.027	0.0083	0.0138^a^	0.976				0.0052
	220°C	235^a^	0.993^a^	0.079	0.0394	0.0684^a^	0.935				0.0173
10:1		Arrhenius analysis (including formic acid)									
	E (kJmol^**−**1^)		149^a^	95(51, 4.1)	109 (^, 22.7)	166^a^					105 (80, 6.4)
	R^2^ of Arrh fit			0.998	0.958						0.996
4:1		Kinetic modelling (including formic acid)									
		235^a^	0.219^a^	0.033	0.055	0.031^a^	0.981				0.0061

Single or first number in brackets, ±95% confidence limits of parameter estimation; second number in brackets, ± standard error of parameter estimation. ^a^Unchanged from results with K_f_ = 0. ^Above high limit.

From Table 3, the values of the 95% confidence limits of the Arrhenius parameter estimates are high, as a result of the limited number of temperature points, although standard errors are acceptable. Comparison of values must therefore be carried out with caution, although with this in mind, the results of the kinetic analysis are broadly consistent with those from previous studies [5]. The activation energy for oligomer release appears to be higher than that for subsequent hydrolysis of oligomers to monomers, which may be a result of the intermolecular hydrogen bonding and steric constraint of hemicellulose polymer within the cell wall matrix. The current analysis also confirms that furan degradation (k_4_) is an important onward pathway, which from the current work appears to have a relatively high activation energy, so becomes increasingly significant at higher process temperatures. This may limit the ultimate concentration in hydrolysate liquors, which will impact on the level of inhibitory contributions of furans in subsequent fermentations. Model studies using xylose have also identified a limit in furfural concentration, which has been interpreted as a result of competing reactions for xylose degradation, rather than furfural instability [15]. However, such model studies will not be influenced by catalytic contributions from the generation of acetic acid from hemicellulose, as discussed later, which may accelerate the rate of furfural breakdown [30]. Also, it is likely that in true biomass mixtures there will be many opportunities for furfural to react with other labile groups or reactive intermediates derived from cell wall components, providing other pathways for its degradation. Such pathways will be of increasing significance as reaction severity is increased, helping to explain the higher activation energy and rate constants than observed for model degradation of furfural [15,19].

### Generation of acetic acid

Acetic acid is believed to be formed as a result of proton catalysed hydrolysis of pendant acetate groups attached at the hydroxyl positions of arabinoxylan polysaccharide [14]. This is consistent with the finding that in water the pH of the reaction liquor typically falls from pH 7 to around pH 4 after treatment. In keeping with this mechanism, the data could be fitted successfully to a first order exponential equation (8), where Ac is the concentration of acetic acid, with fitted parameters summarised in Table 3.

(8) $$Ac_{t}=Ac_{\inf}\left(1-e^{-\mathrm{kt}}\right)$$

The calculated activation energy of 104 kJmol^−1^ is consistent with ester hydrolysis, although at all temperatures the measured rate constants for acetic acid formation were at least five times lower than the corresponding rate constants for solubilisation of hemicellulose. Deacetylation must therefore take place by hydrolysis of acetate groups of xylan oligomers that are already in solution, possibly as well as on intact hemicellulose structures in the cell wall. However, the rate profiles for acetic acid formation were insufficiently resolved to show any evidence of deviation from simple exponential behaviour, which might indicate more than one environment. The relatively slow rate of acetic acid generation suggests that any reduction in pH that this entails will have limited catalytic effect on the kinetics of primary solubilisation, which must therefore occur as a result of true autocatalysis by water. However, the reducing pH may influence catalysis of slower secondary reactions of saccharides in solution.

Fitting of the experimental profiles for generation of acetic acid was achieved with an almost constant initial acetate concentration, of 30 to 32 g/kg (as the acid) on a total biomass basis, at all experimental temperatures. In molar terms, this corresponded to a degree of substitution (DS) on the xylan units of hemicellulose of around 0.44, from the analysed sugar composition of the straw, or 0.36 if substitution is possible on all sugar monomers. This is higher than the DS values of around 0.2 to 0.3, which have been found by analytical hydrolysis of the Hustler variety of wheat straw [31]. Similar molar equivalents of acetic acid were detected in pretreatment hydrolysates of straw by other workers [32].

**Figure 4:**
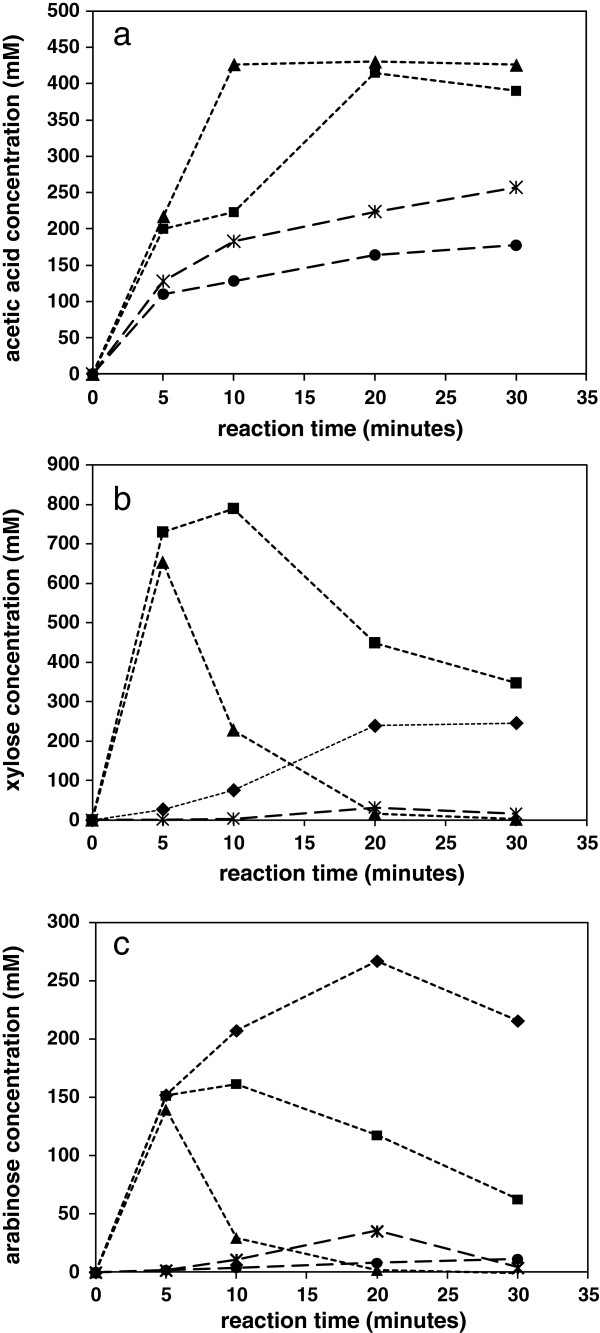
**Evolution of soluble products formed following acid catalysed hydrothermal reaction of cold alkali hemicellulose extract from wheat straw. a)** Acetic acid; **b)** xylose; and **c)** arabinose. Reaction in water: ●, 180°C; *, 200°C. Reaction in 1 wt% aqueous H_2_SO_4_: ♦, 160°C; ■, 180°C; ▲, 200°C.

The trends in acetic acid concentrations in treatment liquors have also been studied by following the hydrothermal reactions of hemicellulose separated from straw, obtained by extraction as detailed in the Methods section. Kinetic data using the microwave reactor at temperatures of 180 and 200°C are shown in Figure 4a, for reactions in water and also in solutions of 1% sulphuric acid. With water as a reaction medium the concentration of acetic acid continued to evolve beyond the experiment time limit, mirroring the data for the whole straw. However, in the presence of the dilute acid catalyst the rate of evolution was faster, as expected, reaching an asymptote of similar concentration at both reaction temperatures. This supports the conclusion that a finite number of acetate substituents on hemicellulose are available for hydrolysis, with no suggestion of continuing liberation of acetic acid due to breakdown of terminal or monomer sugar groups [16]. This allows some confidence in estimation of the total potential acetic acid concentrations, which might be expected in hydrothermal process or fermentation liquors.

From this same series of hemicellulose reactions it was observed that in water the rate of appearance of xylose monomers in the reaction liquor was slow, in Figure 4b, with most solubilised hemicelluloses remaining in oligomer form. The reaction in 1% sulphuric acid was faster, with the hydrolysis of glycosidic bonds leading to a pronounced increase in concentration of xylose monomers with time, in Figure 4b, up to a maximum depending on reaction temperature, followed by a reduction in concentration as degradation reactions became more pronounced. The concentration of arabinose followed a similar set of profiles, in Figure 4c, although at lower overall concentrations, reflecting the lower arabinose content of the hemicellulose. However, under equivalent conditions it was noted that arabinose appeared in solution at a faster rate than xylose, presumably due the ease of cleaving of the single α1-2 or α1-3 arabinose linkages to the xylan backbone. Degradation of the less stable furanose ring was also at a faster rate under equivalent conditions. If the hydrolysis under acid conditions is considered to lead to the most quantitative generation of monomers, then making estimations from the data for degradation losses, the comparison of molar concentrations in the reaction liquor suggests the degree of acetate substitution on the original extracted arabinoxylan of around 0.36, including xylose and arabinose but excluding other minor saccharides. This is in line with the amount of acetate determined from the hydrothermal treatment of the whole straw.

### Generation of formic acid

Formic acid is also detected in significant amounts in hydrothermal hydrolysates, although its origins are less well understood, with possible mechanisms associated with the degradation of furan species [16,29]. However, the kinetic profiles in Figure 2c reveal that in molar terms formic acid is released under these hydrothermal conditions at a faster rate than production of furfural, especially at early times, so may not be generated purely by furfural breakdown. The apparent first order behaviour is also inconsistent with this reaction pathway. However, an alternative mechanism for the generation of formic acid, which may be consistent with the data, involves the direct breakdown of a saccharide reducing end group, which is first rearranged to form the 1–2 endiol, and which then hydrolyses to liberate formic acid [33]. This mechanism would require an early availability of saccharide end groups in the hydrolysate liquor, which would come from hemicellulose fragments including oligomers and hydrolysed substituents.

**Figure 5:**
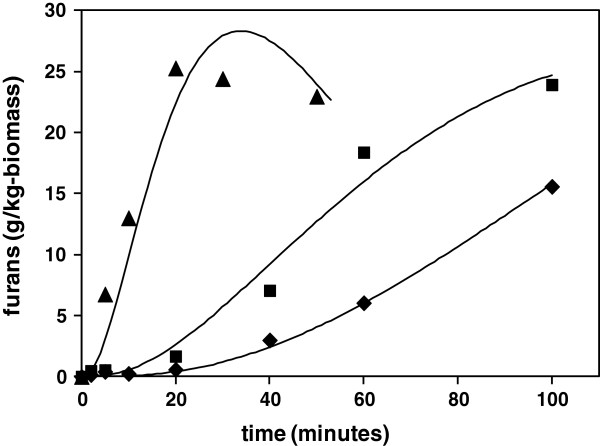
**Simulation of kinetics of generation of furans, with inclusion of positive rate constant for formic acid generation from soluble hemicellulose.** ♦, 180°C; ■, 200°C; ▲, 220°C.

The pseudo-first order kinetic behaviour shown by formic acid may therefore conceal a more complicated reaction scheme, which may involve competition with the main pathway for generation of furfural. If it is assumed that formic acid can be formed by end group reactions of solubilised hemicellulose then this will result in a split in the k_2_ pathway from this point, which may be introduced into the scheme by inclusion of an additional parallel first order reaction with a rate constant k_fa_, as shown in Figure 3. A corresponding additional term is included in the differential equation (5). Attempts at calculation of formic acid kinetic profiles with this revised scheme were quite successful, with fitted data shown as continuous lines in Figure 2c, with parameters summarised in Table 3. The pseudo-first order behaviour then comes from the rapid solubilisation of hemicellulose, giving an apparent fixed reagent concentration. Subsequent adjustment of the other rate constants in the analysis were required to maintain good fits of furfural profiles, which led to a reduction in k_2_ for monosaccharide formation and an increase in k_3_ for furfural formation, with the rate constant k_4_ for furfural degradation unchanged. The alternative rate parameters including k_fc_ are summarised in Table 3. The alternative fits to the furan experimental profiles are shown in Figure 5, which are acceptable for data at 180 and 200°C, but slightly less so at 220°C. However, overall the scheme is still reasonable as an explanation of formic acid generation from polysaccharide containing biomass under these hydrothermal conditions.

### The influence of liquor content

**Figure 6:**
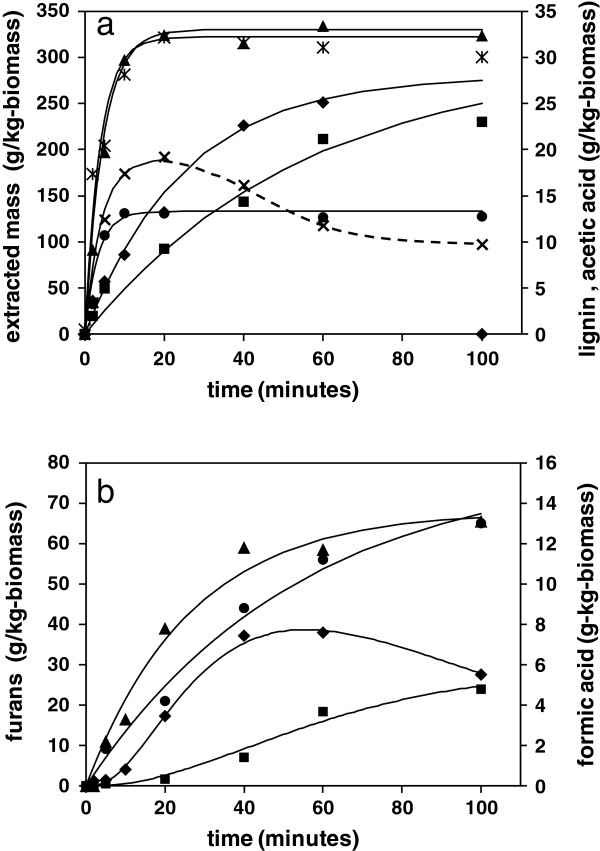
**Influence of liquor ratio on kinetics of inhibitor formation following hydrothermal reactions at 200°C. a)** Primary reactions of total solubilised mass: *, 4:1; ▲, 10:1; soluble lignin: x, 4:1; ●, 10:1; and acetic acid: ♦, 4:1; ■, 10:1. **b)** Secondary reactions of furans: ♦, 4:1; ■, 10:1; and formic acid: ▲, 4:1; ●, 10:1.

A further series of hydrolysate liquors were collected from hydrothermal reactions at a temperature of 200°C, at a lower water to biomass ratio of 4:1. The UV measurement of the liquors was again used and provided an indication of the evolution of the key soluble lignin and furanic compounds over time, shown in comparison with the corresponding 10:1 liquor ratio data in Figure 6. From the gravimetric weight loss determinations, in g/kg-dry biomass units, it appeared that the global solubilisation reactions proceeded at the same rate at the lower liquor ratio, within the limits of the experimental resolution, releasing the same amount of biomass material. The overall rate of lignin solubilisation was similar at both 4:1 and 10:1 liquor ratios, but a greater amount of lignin was apparently solubilised at lower liquor ratio. There was also a noticeable reduction in soluble lignin concentration as the reaction proceeded to longer times, which is presumed to be due to an increased likelihood of recondensation of reactive lignin species at higher biomass concentration. This was also mirrored in a slight reduction in overall weight loss at longer reaction times.

Acetic acid was apparently liberated at a higher rate at the lower 4:1 liquor ratio, from Table 3, but still at a lower rate than that of the solubilisation of the hemicellulose polysaccharides. However, it is possible that the faster generation of additional protons and the reduction in absolute pH has induced to an additional autocatalytic influence on the ester hydrolysis reactions. The development of furan species may also be accelerated at lower liquor ratio for the same reason with Figure 6 showing a maximum furan concentration reached at earlier times and at a higher level than at 10:1 liquor ratio. The same catalytic influences may also help to explain the faster rate of formation of formic acid, with the kinetic constants from the simulation summarised in Table 3.

### Implications and limitations of the kinetic analyses

Overall, it is hoped that the kinetic analyses of this study will assist in understanding the key reactions resulting from hydrothermal deconstruction of biomass and may also assist with the development of improved cell wall structure–property relationships. The scheme of kinetic analyses is less complex than other published models and the methodology has been chosen as the simplest effective approach for describing the series of changing concentrations of reaction products [34]. The microwave technique allows the determination of species concentrations at accurately defined temperatures and times, providing additional insights into the relationships and interconnections between the various deconstruction processes. The tools used in this work to perform numerical integration of coupled ODEs have not permitted the determination of confidence limits for the k_2_, k_3_ and k_4_ constants of the kinetic scheme, and also the sparseness of rate constant data points leads in some instances to high confidence limits for activation energies. However, the return of high confidence limits does not necessarily invalidate the best fit parameter values [34].

This work has shown that flexibility in choice of reaction conditions will be helpful in minimising inhibitor release, although other release factors relate to the intrinsic chemical nature of the cell wall polymers. The work shows that the liberation of soluble lignin species occurs at a very similar rate to the reactions leading to hemicellulose depolymerisation, so may be related to the same deconstruction processes in the cell wall. Hence, a reduction in production of soluble aromatic inhibitors will result in a reduced overall extent of deconstruction, which will need to be counterbalanced by achieving a higher effectiveness of subsequent enzyme digestion. The work also shows that the rate of acetate release is somewhat slower than that of the global progress of deconstruction, so the increase in acetic acid concentration might also be minimised by avoiding an over-long reaction time. The selection of a compromise reaction time would also be beneficial in minimising the secondary generation of furan species. However, the work does show that the level of furan and lignin-derived inhibitors are likely to reach a maximum as further reactions induce either recondensation or breakdown into smaller species. This might place a limit on the required chemical tolerance of yeast. There will also be a limit on the level of tolerance required towards acetic acid, when all acetate groups are hydrolysed from the hemicellulose. However, the operation at excessive reaction times or increased severity is undesirable, as sugar yield will be reduced and concentrations of toxic end products such as of formic acid will be increased. Operation at lower liquor ratio is desirable to maximise sugar concentrations for subsequent enzyme hydrolysis and fermentation, but this is seen to accelerate the rate of generation of some inhibitor compounds.

The knowledge of degradation pathways may provide further direction to programmes concerned with optimisation of phenotypic characteristics of biomass for highest process efficiencies. The hemicellulose of the wheat variety used in the study is quite highly acetylated, which will exaggerate the difficulty in minimising the concentration of acetic acid in process liquors. The selection of varieties with lower acetyl content in the cell wall could therefore be beneficial from a process perspective. Likewise, the production of formic acid may be more prevalent in hydrolysates from plant species containing hemicelluloses with greater amounts of labile substituents such as arabinose. Selection for a low arabinose or an overall less decorated hemicellulose as a constituent of the cell wall may be beneficial in limiting the generation of all sugar-derived inhibitors, including both organic acids and furans, allowing a wider range of deconstruction process conditions.

The kinetic information allows a consideration of the most favourable overall reaction conditions in a hypothetical process, striking a compromise between the necessary biomass deconstruction and the undesired liberation of inhibitor compounds. From the primary kinetics of solubilisation, it is concluded that at a reaction temperature of 200°C the major deconstruction reactions are complete by 20 minutes, which is a practical residence time in a continuous system (4). At this residence time the liberation of soluble lignin has unavoidably reached a maximum, but other inhibitors, including formic acid, acetic acid and furanic compounds, are at concentrations below their eventual maximum. From the current model, the inhibitor concentrations at this time interval are summarised in Table 4 for the two liquor ratios considered. Clearly real-life optimisation would need to take account of many other factors, defined by a full optimisation problem, including mixing, chemical diffusion and thermal diffusion, and energy/efficiency trade-offs.

**Table 4:** Potential concentrations of inhibitor compounds formed at 20-minute reaction times at 200°C under hydrothermal conditions

**Species**	**Molecular weight**	**Liquor ratio = 10:1**		**Liquor ratio = 4:1**	
		**g/kg**	**mM**	**g/kg**	**mM**
Lignin	188^a^	13	6.9	18	9.5
Acetic acid	60	9	15	16	27
Furans	96^b^	2.5	2.6	18	19
Formic acid	46	5	10.9	8.5	18.5

^a^Vanillic acid; ^b^furfural.

## Conclusions

The use of a microwave synthesis reactor has allowed kinetic data for the hydrothermal reactions of straw biomass to be established from short times, avoiding corrections required for slow heating in conventional reactors, or two-step heating. The gravimetric loss through solubilisation of straw provides a global measure of the extent of deconstruction, giving rise to an Arrhenius activation energy of 148 kJmol^−1^, which is higher than activation energies used historically for derivation of empirical measures of reaction severity. The gravimetric loss is primarily due to depolymerisation of the hemicellulose component of straw, but a minor proportion of lignin is solubilised at the same rate and hence may be associated with the more hydrophilic lignin-hemicellulose interface. Acetic acid is liberated primarily from hydrolysis of pendant acetate groups on hemicellulose, although the rate is too slow to provide catalytic enhancement to the primary solubilisation reactions. However, the increase in acidity may enhance secondary reactions leading to the production of furans and formic acid. The work has suggested that formic acid may be formed under these hydrothermal conditions via direct reaction of sugar end groups rather than furan breakdown. Furan degradation reactions are found to be significant, which may limit ultimate concentrations of furans in hydrolysate liquors.

## Materials and methods

### Samples

Wheat straw (Zebedee variety), comprising stem and leaf components, was provided by the University of Nottingham farm, Nottingham, UK, which was stored under dry conditions after harvesting for approximately 3 months prior to use. All samples were knife milled to a 2 mm mesh size (Pulverisette 19; Fritsch GmbH, Idar-Oberstein, Germany), which was a form suitable for hydrothermal treatments. Prior to investigations, samples were conditioned to equilibrium moisture content in the ambient laboratory environment. Gravimetric moisture determinations of all as-received materials were carried out, with subsequent analytical results quoted on a dry mass basis. A separate sample of wheat straw was swelled in 4 M potassium hydroxide over 2 hours at ambient temperature at a liquor to solid ratio of 20:1, in order to extract a hemicellulose-rich fraction. The extraction liquor was diluted in water, neutralised with acetic acid and then the soluble products were precipitated by addition of acetone. The precipitate was filtered and rinsed in aqueous ethanol before vacuum drying, followed by weighing to provide an estimate of total hemicellulose content.

### Analysis of straw composition

Total acid hydrolysis of the as-received wheat straw for sugar analysis was carried out by immersion in 12 M sulphuric acid for 2 hours at 35°C, then 1 M sulphuric acid for 2 hours at 98°C [9]. Analysis of soluble sugar monomers was by high-performance anion exchange chromatography with pulsed amperometric detection (Dionex, Camberley, UK), using a CarboPac PA20 column under isocratic conditions, with 10 mM NaOH as the mobile phase at a working flow rate of 0.5 ml/min. Glucose, xylose, arabinose and galactose were used as standards with mannitol as internal standard. Analysis of lignin in the as-received straw was carried out by extraction using acetyl bromide in water/dioxane solvent, followed by measurement of absorbance at 280 nm [9]. Lignin quantification was performed by calibration using a low sulphate lignin reference material. The amount of organic extractable material in as-received straw was determined by Soxhlet extraction for 18 hours in pure ethanol. The extracted mass was determined by weight after rotary evaporation of the liquor followed by vacuum drying [35]. All compositional values were calculated on a dry weight basis, summarised in Table 1.

### Hydrothermal reactions

A bench-top microwave reactor was used for all hydrothermal processing (Monowave 300; Anton Paar, St Albans, UK). One gram amounts of as-received dry biomass were added to each 30 ml glass reactor vial, into which was decanted either 10 or 4 ml of demineralised water (liquor ratio of 10:1 or 4:1). The mixture was shaken to ensure full wetting of the biomass particles, then stood for 1 hour before carrying out reactions. Vials were sealed with plastic/silicone caps, which were fitted with an insert for a ruby luminescence thermometer positioned with the tip centrally located within the biomass material. Vials were loaded sequentially into the reactor, which was programmed for fastest possible heating up to the chosen set temperature, either 180, 200 or 220°C. The instrument delivered a dynamic power profile giving a smooth heat-up in a time of around 80 ± 10 seconds for all temperatures, accurately controlled from the ruby luminescence temperature sensor. The heat-up phase was followed by a controlled isothermal period maintained at lower power, with a temperature accuracy of ±0.5°C, which was followed by rapid cooling by forced air circulation around the vial. The reactor was opened at a safe temperature of less than 60°C. An equivalent set of assay experiments were carried out using the hemicellulose-rich solid extracted from the straw, at reaction temperatures of 180 and 200°C in an aqueous solution of 1% (w/w) sulphuric acid.

### Determination of solubilised mass

Following the hydrothermal reaction, the vials with the 10:1 liquor ratio samples were shaken by hand and allowed to stand for 15 minutes, before vacuum filtering through a preweighed glass filter paper. The filtered hydrothermal liquor (hydrolysate) was collected for analysis and then further repeated quantities of water were rinsed through the remaining filter residue, to ensure full removal of all water soluble products. The filter paper and residue were then allowed to dry in the ambient laboratory atmosphere overnight. Finally, all filter papers were dried at 105°C for 2 hours in an air circulation oven, then weighed to establish the retained weight of the solid straw residue and hence the weight loss through solubilisation during the hydrothermal reaction. Independent measurement of replicate samples indicated that the standard error of weight loss determinations was around ±6 g/kg. The same filtration and washing procedure was used for the 4:1 liquor ratio samples except that 6 ml of distilled water was added to the vials after removal from the reactor, to make them up to the same volume as the 10:1 liquor ratio samples.

### Analysis of hydrothermal liquors

Analysis of the total amount of solubilised lignin in the separated hydrothermal liquors was carried out by measurement of the UV absorbance at 320 nm, according to a published method [36]. Analysis of the total amount of furanic compounds in the hydrothermal liquors was also determined by measurement of UV absorbance, at 280 nm, with subtraction of the intensity at 320 nm, to account for spectral overlap with lignin [37]. Independent measurements indicated that the standard error of lignin determination was around ±0.4 g/kg. Determination of acetic and formic acids was performed by HPLC using a Rezex ROA-Organic Acid H + column, at ambient temperature, with 0.005 N H_2_SO_4_ mobile phase at a flow rate of 0.5 ml/min, with UV detection. The standard error of measurement was around ±0.6 g/kg. Confirmatory analyses of furfural and HMF were also carried out by the Rezex chromatographic method, to verify the robustness of the UV method for total furans. The sugar monomer concentrations in the hydrolysates from the separate reactions of the extracted hemicellulose-rich solid were analysed using the anion exchange chromatography method, as above. The concentrations of acetic acid in the hemicellulose hydrolysates were determined using the Rezex method as described.

### Data analysis

Fitting of simple first order integral rate equations (1 and 8) was carried out using GraphPad Prism (GraphPad Software, Inc, San Diego, CA, USA), including reporting of 95% confidence intervals of rate constant parameters. Arrhenius analysis was also carried out using GraphPad Prism, including reporting of both 95% confidence intervals and standard errors of parameter estimates. For the linked kinetic scheme in Figure 3 the calculation of species concentrations was performed by numerical integration of the coupled ODEs (4, 5, 6 and 7) using a step method in Microsoft Excel (Microsoft, Redmond, WA, USA). Mass conservation by this method was confirmed. Error minimisation between calculated and experimental concentrations was carried out using the Excel Solver Add-in. Also, R^2^ correlation coefficients were calculated from the squared residuals between calculated and experimental data points. Calculation of confidence intervals was not possible via the Excel method.

## Abbreviations

DS: Degree of substitution; HMF: 5-(Hydroxymethyl)furfural; HPLC: High performance liquid chromatography; LR: Liquor to solid ratio; ODE: Ordinary differential equation; UV: Ultraviolet spectroscopy.

## Competing interests

The authors declare that they have no competing interests.

## Authors’ contributions

All authors contributed to the conception and design of the work. Physical treatments and kinetic analyses were carried out by RI. Chemical analyses were carried out by SG. Cell wall structures and models were developed with the support of GT. Pretreatment methodologies were developed with the support of SH. Inhibitor schemes were developed with the support of DG. All authors contributed to the drafting and revision of the manuscript, and approved the text and diagrams for submission.
